# Abnormal Intrinsic Brain Activity Patterns in Patients with Subcortical Ischemic Vascular Dementia

**DOI:** 10.1371/journal.pone.0087880

**Published:** 2014-02-03

**Authors:** Chen Liu, Chuanming Li1, Xuntao Yin, Jun Yang, Daiquan Zhou, Li Gui, Jian Wang

**Affiliations:** 1 Department of Radiology, Southwest Hospital, Third Military Medical University, Chongqing, China; 2 Department of Neurology, Southwest Hospital, Third Military Medical University, Chongqing, China; University of Manchester, United Kingdom

## Abstract

**Objectives:**

To investigate the amplitude of low-frequency fluctuations (ALFF) alteration of whole brain in patients with subcortical ischemic vascular dementia (SIVD).

**Materials and Methods:**

Thirty patients with SIVD and 35 control subjects were included in this study. All of them underwent structural MRI and rs-fMRI scan. The structural data were processed using the voxel-based morphometry 8 toolbox (VBM8). The rs-fMRI data were processed using Statistical Parametric Mapping (SPM8) and Data Processing Assistant for Resting-State fMRI (DPARSF) software. Within-group analysis was performed with a one-sample Student's *t*-test to identify brain regions with ALFF value larger than the mean. Intergroup analysis was performed with a two-sample Student's *t*-test to identify ALFF differences of whole brain between SIVD and control subjects. Partial correlations between ALFF values and Montreal Cognitive Assessment (MoCA) and Mini-Mental State Examination (MMSE) scores were analyzed in the SIVD group across the parameters of age, gender, years of education, and GM volume.

**Results:**

Within-group analysis showed that the bilateral anterior cingulate cortex (ACC), posterior cingulate cortex, medial prefrontal cortex (MPFC), inferior parietal lobe (IPL), occipital lobe, and adjacent precuneus had significantly higher standardized ALFF values than the global mean ALFF value in both groups. Compared to the controls, patients with SIVD presented lower ALFF values in the bilateral precuneus and higher ALFF values in the bilateral ACC, left insula and hippocampus. Including GM volume as an extra covariate, the ALFF inter-group difference exhibited highly similar spatial patterns to those without GM volume correcting. Close negative correlations were found between the ALFF values of left insula and the MoCA and MMSE scores of SIVD patients.

**Conclusion:**

SIVD is associated with a unique spontaneous aberrant activity of rs-fMRI signals, and measurement of ALFF in the precuneus, ACC, insula, and hippocampus may aid in the detection of SIVD.

## Introduction

Dementia is a syndrome with numerous symptoms, including loss of memory, judgment, reasoning, and changes in mood, behavior, and communication [1]. Vascular dementia (VaD) is the second most common type of dementia after Alzheimer disease (AD), and it accounts for up to one third of all dementias [2]. In contrast to AD, the cognitive profile of VaD includes prominent executive dysfunction and attention deficiency, but less prominent memory impairment [3]. VaD encompasses multiple vascular pathologies and are divided into several subtypes based on the nature of the vascular disease and the clinical manifestations [3]. Subcortical ischemic vascular dementia (SIVD) is a relatively homogeneous subtype of VaD in terms of lesion location and clinical manifestations and is characterized by the presence of lacunar infarcts and white matter lesions, which are both mediated by small vessel disease [4].

The recent development of resting-state functional MRI (rs-fMRI) has allowed researchers to detect intrinsic brain activity during rest and has provided valuable insights into the pathomechanism of dementia [5], although currently it can't exclude the vascular effects which may contribute to the rs-fMRI signal. The amplitude of low frequency fluctuations (ALFF) was recently proposed to assess the amplitude of resting-state spontaneous brain activity by calculating the square root of the power spectrum in a frequency range (typically 0.01–0.08 Hz) [6], [7]. Decreased ALFFs in the medial parietal lobe and increased ALFFs in the lateral temporal, frontal, and parietal regions, which show significant correlations with cognitive performance measured by the Mini-Mental State Examination (MMSE) have been found in AD and MCI patients [8], [9]. In this study, we observed and analyzed the ALFF alterations of whole brain in SIVD patients and examined their correlations with cognitive performance to enhance our understanding of the pathomechanism of SIVD.

Moreover, recent studies have suggested that ALFF results may be affected by structural atrophy [10]. Because decreased gray matter (GM) volume in the frontal and temporal regions as well as the hippocampus and anterior cingulate cortex has been observed in SIVD patients [11], [12], to reduce the effect of brain atrophy on ALFF measurements, we additionally performed an analysis of GM volume and used this information as a covariate in our ALFF analysis.

## Materials and Methods

### Ethics Statement

All research procedures were approved by the Institutional Review Board of the Third Medical Military University and were conducted in accordance with the Declaration of Helsinki. The individuals in this manuscript have given written informed consent (as outlined in PLOS consent form) to participate in this study and publish these case details. Because cognitive disability can make it impossible to obtain valid informed consent, we also acquired written informed consent from the patients' surrogates (spouse or child). All potential participants who declined to participate or otherwise did not participate were eligible for treatment (if applicable) and were not disadvantaged in any other way by not participating in the study.

### Subjects

Thirty-four consecutive SIVD patients presenting to our institution between December 2010 and September 2012 were enrolled in this study (Table 1). All patients were required to complete a neurologic interview to determine their history of onset, symptoms, and recovery from stroke as well as memory loss and other symptoms of dementia. Clinical diagnosis of SIVD was made by a senior neurologist according to the criteria made by Erkinjuntti [13] and the DSM-IV (Diagnostic and Statistical Manual of Mental Disorders, IV). Examination included the following tests: MMSE, Clinical Dementia Rating (CDR), Neuropsychiatric Inventory (NPI), Activities of Daily Living Scale (ADL), Hachinski and Hamilton test, Wechsler Memory Scale, verbal and categorical fluency, Figural Recognition Test, and Montreal Cognitive Assessment (MoCA). Standard clinical CT or MRI scanning during hospitalization were performed to diagnose subtypes of stroke and exclude cerebral hemorrhage. The exclusion criteria included the following: patients with aphasia and patients who otherwise could not complete the psychological tests because of a language disorder, diagnosis with a chronic or degenerative disease, active substance abuse disorders, intracranial hemorrhage, difficulty in controlling epilepsy that could present with cognitive impairment, and temporal lobe epilepsy. However, past or present medication for the treatment of cognitive impairment and infarction was not an exclusion criterion. Thirty-five age and gender matched healthy individuals were recruited from the local community. All of them undergone neurological and neuropsychological evaluation and conventional magnetic resonance imaging (MRI), and was confirmed as a control subject by an MMSE score of 27 or greater, and a MoCA score of 26 or greater. None of them had vascular risk factors, cognitive complaints, current psychiatric illness or history of psychiatric illness.

**Table 1:** Demographic and clinical data of SIVD patients and control subjects.

Characteristics	SIVD patients (*n* = 30)	Control subjects (*n* = 35)	*P*-value
**Gender (male/female)**	19/11	22/13	0.97^a^
**Age (years)**	57–85(69.0±7.8)	58–83(68.0±5.8)	0.56^b^
**Education (years)**	0–18(7.5±4.7)	3–17(9.2±3.3)	0.10^b^
**MMSE**	4–23(16.1±5.1)	27–30(28.4±1.1)	<10^−5b^
**MoCA**	2–14(9.4±3.8)	26–30(27.2±1.5)	<10^−4b^
**HIS**	8–17(10.2±1.9)	—	
**GDS**	3–7(4.8±1.0)	—	
**CDR**	1–3(1.6±0.7)	—	
**ADL**	27–80(49.9±16.5)	—	

Data were expressed as the range from min – max (mean ± SD). Abbreviations: MMSE, Mini-Mental State Examination; MoCA, Montreal Cognitive Assessment; HIS, Hachinski Ischemic Score; GDS, Global Deterioration Scale; CDR, Clinical Dementia Rating; ADL, Activities of Daily Living Scale.

a *P*-value was obtained using the two-tailed Chi-squared test.

b *P*-value was obtained using the two-sample, two-tailed *t*-test.

### MR Image Acquisition

All participants were scanned using a 3T scanner (MAGNETOM Trio Tim System, Siemens, Erlangen, Germany) with a 12-channel head coil. Head motion was restricted with foam padding around the head, and the importance of head immobility was explained to each subject. Conventional MRI included transverse fluid-attenuated inversion recovery (FLAIR) (TR/TE/TI = 9000/93/2500 ms, flip angle = 130°, matrix = 256×256, thickness = 4.0 mm, 25 slices, voxel size = 0.9×0.9×4 mm^3^) and T_1_-weighted images (TR/TE/ = 200/2.78 ms, flip angle = 70°, matrix = 384×384, thickness = 4.0 mm, 25 slices, voxel size = 0.7×0.6×5 mm^3^). Resting-state functional images were acquired using echo-planar imaging (EPI) sequence with the following parameters: TR/TE = 2,000/30 ms, flip angle = 90°, matrix = 64×64, thickness = 3 mm, gap = 1 mm, 36 slices, voxel size = 3.5×3.5×3.0 mm^3^. Structural images were acquired using Magnetization-prepared rapid gradient echo (MPRAGE) sequence with the following parameters: TR/TE/TI = 1, 900/2.52/900 ms, flip angle = 9°, matrix = 256×256, thickness = 1.0 mm, no gap, 176 slices, voxel size = 1×1×1 mm^3^.

### Image Processing and Analysis

For clinical imaging data analysis, the location/number of the infarcts and the grade of white matter lesions (WML) (grade 0, absent; grade 1, punctate; grade 2, early confluent; grade 3, confluent; according to the grading scale presented by Fazekas [14]) were assessed on FLAIR images by agreement among three neurologists.

For structural data processing and analysis, the voxel-based morphometry 8 (VBM8) toolbox (http://dbm.neuro.uni-jena.de/vbm.html) was used. Images were corrected for bias-field inhomogeneity, registered using linear (12-parameter affine) and nonlinear transformations, and tissues were classified as GM, white matter, or cerebrospinal fluid within the same generative model [15]. The GM images were adjusted to account for volume changes due to normalization. Only nonlinear volume changes were considered, so that differences in head size would not need to be considered. Images were smoothed to a Gaussian kernel of 6-mm full width at half maximum (FWHM). Per-subject GM volume was calculated and used as a covariate in the functional analysis of group differences.

Rs-fMRI data processing was performed using Statistical Parametric Mapping (SPM8, http://www.fil.ion.ucl.ac.uk/spm) and Data Processing Assistant for Resting-State fMRI (DPARSF, http://rest.restfmri.net) software [15]. The first five volumes of each functional time series were discarded because the signals reached equilibrium, and the participants were adapting to the scanning noise. The remaining 235 images were then corrected for the within-scan acquisition time differences between slices and further realigned to the first volume. Head movement parameters were computed by estimating translation in each direction and angular rotation on each axis for each volume. As described in previous study, head motion can significantly influence measures and results derived from the resting-state fMRI scan [16]. Hence, we also examined the group differences of head motion by using two-sample t-tests according to mean framewise displacement (FD) Jenkinson measurement [17], [18]. Four SIVD patients were excluded according to the criteria that individuals must not have an estimated maximum displacement in any direction greater than 2 mm or a head rotation greater than 2°. After motion correction, each individual structural image was coregistered with the mean rs-fMRI image. The transformed structural images were then segmented (New Segmentation) into GM, white matter, and cerebrospinal fluid using a unified segmentation algorithm. A local brain template was generated using diffeomorphic anatomical registration through exponential Lie algebra (DARTEL). Motion-corrected functional volumes were spatially normalized to the Montreal Neurological Institute (MNI) space and resampled to 3-mm isotropic voxels using the normalization parameters estimated during unified segmentation. The normalized rs-fMRI data were smoothed to 6-mm FWHM.

After linear trend removal, the time series was transformed to the frequency domain using a fast Fourier transform (FFT). The power spectrum obtained by FFT was square root transformed and averaged across frequencies of 0.01 to 0.08 Hz. This averaged square root was termed the ALFF. To reduce the global effects of variability across participants, as performed in many positron emission tomography (PET) studies, the ALFF of each voxel was divided by the global mean ALFF value for each subject. The global mean ALFF value was calculated for each participant within the GM mask. The GM mask was defined as the mean GM intensity of all 65 subjects larger than 0.2 [19]. The relative ALFF value in a given voxel reflected the degree of the raw ALFF value relative to the average ALFF value of the whole brain. The grand-mean ALFF value of each subject was also calculated.

### Statistical Analysis

Within-group analysis was performed with a one-sample Student's *t*-test (within the GM mask) to identify brain regions with larger ALFF values than the mean. Intergroup analysis was performed with a two-sample Student's *t*-test to identify the ALFF difference between SIVD and control subjects. Partial correlations between the mean ALFF values of the brain regions that showed inter-group differences and the neurological scales, including the MoCA and MMSE scores, were analyzed. Age, gender, education level, mean FD and grand-mean ALFF values of each subject were imported as covariates in the statistical analysis. Considering that structural differences among groups might affect the functional results [10], the relative ALFF results were reanalyzed with two-sample *t*-tests using the GM volume as a covariate. The statistical thresholds were set at *P*<0.01 and a cluster size larger than 1080 mm^3^, which corresponds to a corrected *P*<0.05 in the AlphaSim program (http://afni.nih.gov/afni/docpdf/AlphaSim.pdf).

## Results

The demographic characteristics and neuropsychological scores were presented in Table 1. Gender, age, and years of education did not differ significantly between the two groups. The MMSE and MoCA scores of the SIVD group were significantly lower than those of the control group (*P*<0.0001). There is no significant mean FD difference between the two groups (0.1748±0.0123 mm for control group; 0.1769±0.0239 mm for SIVD group; t = −1.682, p = 0.10).

The numbers of patients with infarcts were 10 in thalamus/basal ganglia, 6 in frontal, 5 in parietal, 1 in temporal and 1 in occipital lobes. The numbers of patients with WML were as follows: punctate (grade 1) = 7, early confluent (grade 2) = 6, confluent (grade 3) = 21.

**Figure 1:**
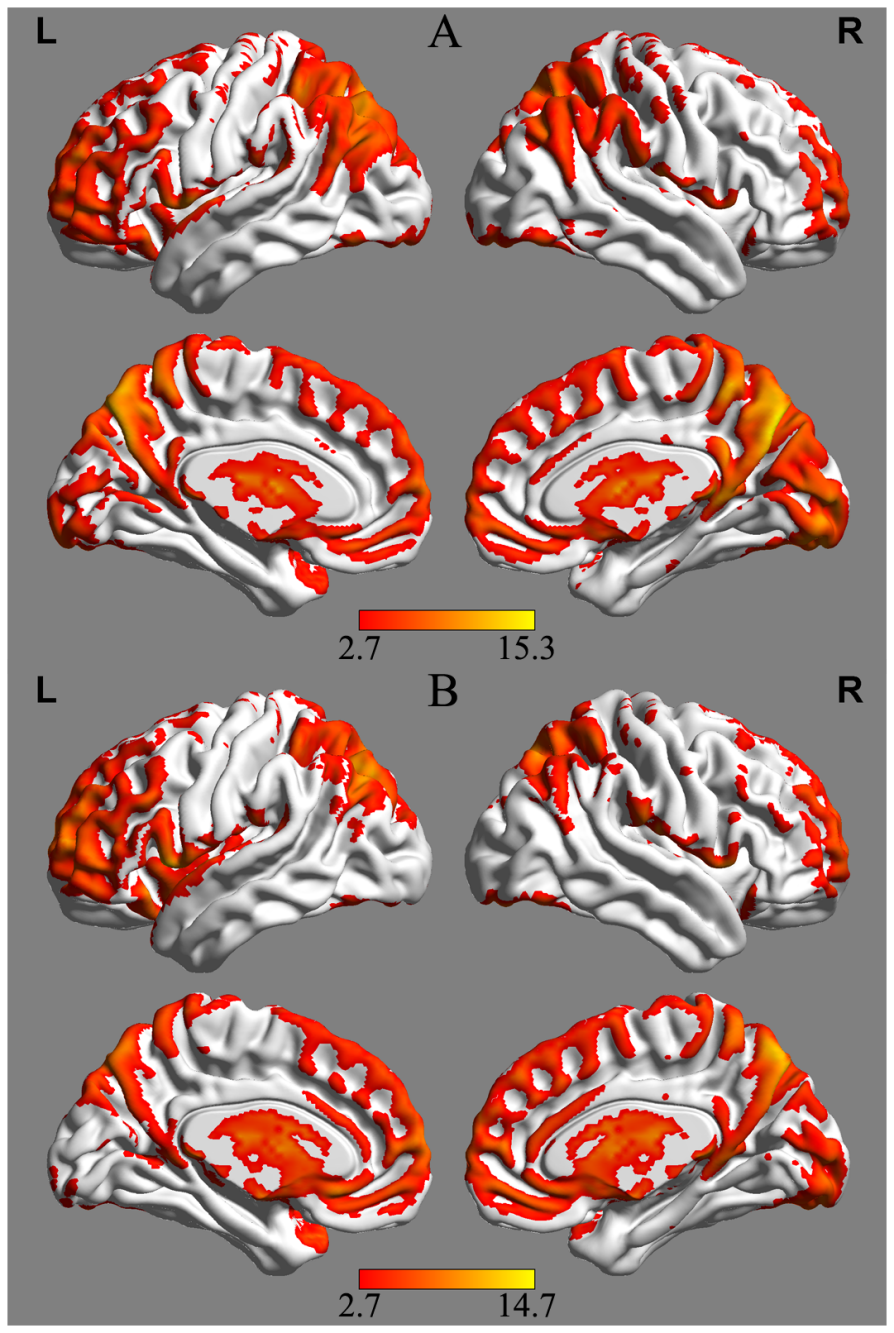
Mean amplitude of low-frequency fluctuation (ALFF) maps from the control group (A) and the subcortical ischemic vascular dementia (SIVD) group (B). Visual inspection indicated high ALFF values in several regions, including the anterior cingulate cortex (ACC), posterior cingulate cortex (PCC), and the adjacent precuneus. The left side of the image denotes the left side of the brain. Color bars represent the *t* value of the group analysis. The t statistical maps were created using the BrainNet Viewer (http://www.nitrc.org/projects/bnv/).

Within-group analysis showed that the bilateral ACC, posterior cingulate cortex, medial prefrontal cortex (MPFC), inferior parietal lobe (IPL), occipital lobe, and adjacent precuneus had significantly higher standardized ALFF values than the global mean ALFF value in both groups. The group-level ALFF maps were then visualized with the BrainNet Viewer (http://www.nitrc.org/projects/bnv/) (Figure 1).

**Figure 2:**
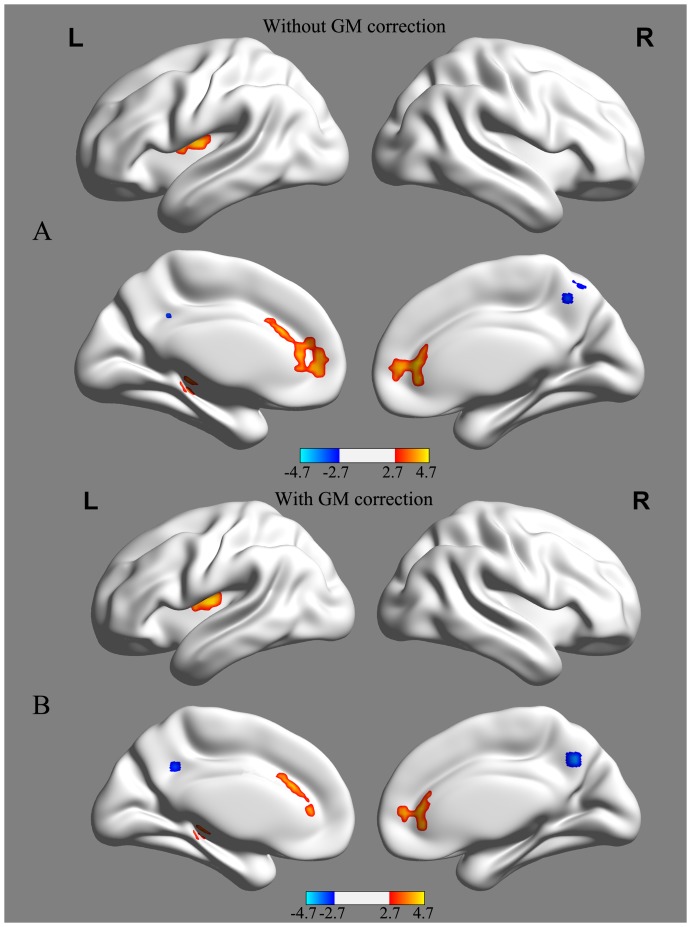
ALFF differences between SIVD and control groups without (A) and with (B) GM volume correction, respectively. SIVD patients showed reduced ALFF values in the bilateral precuneus and increased ALFF values in the bilateral ACC, left insula and hippocampus. Of note, the between-group differences in the ALFF values exhibited highly similar patterns between with and without correcting GM volume. Statistical thresholds were set at *P*<0.01 for individual voxels and a cluster size >1080 mm^3^, which corresponds to a corrected *P*<0.01 determined by Monte Carlo simulations. Color bars represent the *t* value of the group analysis. Cool color represents decreased ALFF values, and warm color represents increased ALFF values. The *t* statistical maps were created using the BrainNet Viewer (http://www.nitrc.org/projects/bnv/).

**Figure 3:**
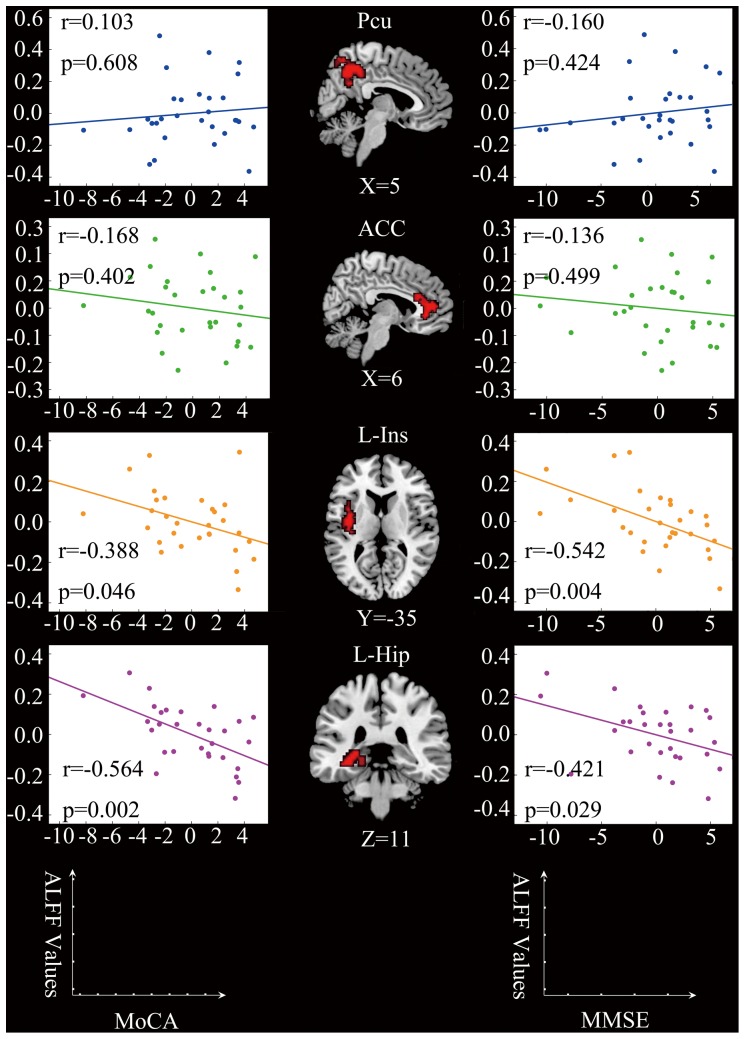
Correlations between ALFF values and cognitive performance of patients with SIVD. Left column: partial correlation analysis between MoCA scores and ALFF values. There were negative correlations between the MoCA scores and the average ALFF values of the left insula (*r* = −0.388, *p* = 0.046) and the left hippocampus (*r* = −0.564, *p* = 0.002). Middle column: regions showing differences in ALFF values between SIVD patients and control subjects (SIVD>HC, *p*<0.01, AlphaSim corrected). Right column: partial correlation analysis between MMSE scores and ALFF values. There was negative correlation between the MMSE scores and the average ALFF values of the left insula (*r* = −0.542, *p* = 0.004) and the left hippocampus (*r* = −0.421, *p* = 0.029). The effects of age, gender, years of education, and GM volume were corrected as covariates. Each dot represents data from one participant.

Compared to the controls, patients with SIVD presented decreased ALFF values in the bilateral precuneus and increased ALFF values in the bilateral ACC, left insula, and left hippocampus. There was no significant difference between two groups (0.6593±0.1921 for control group; 0.6993±0.2035 for SIVD group; t = −0.815, p = 0.42) in grand-mean ALFF values. When using GM volume as an additional covariate, although the amplitudes of the t statistic and the cluster size decreased slightly, the ALFF group difference exhibited highly similar spatial patterns to those for which the GM volume was not corrected (Table 2, Figure 2). This suggests that differences in ALFF values were not a total result of regional atrophy. Negative correlations were found between the ALFF values of the left insula and the left hippocampus and both the MoCA and MMSE scores of SIVD patients (Figure 3). No significant correlation was found between the ALFF values of the ACC and the precuneus and both MMSE and MoCA scores of SIVD patients.

**Table 2:** Regions showing ALFF differences between SIVD and control groups.

			MNI coordinate (mm)			
Brain regions	BA	Number of cluster voxels	X	y	z	Maximum T
**Decreased ALFF in SIVD**						
**Bilateral PCu**	7	83	3	−54	48	−3.68
**Increased ALFF in SIVD**						
**Bilateral ACC**	32	197	6	36	0	4.57
**Left Ins**	13	50	−39	−9	12	4.25
**Left Hip**	27	61	−33	−33	−6	4.02

Note: Comparisons were performed at *P*<.01, corrected for multiple comparisons using AlphaSim program. X, y, z, coordinates of peak locations in the MNI space. Maximum *t*, statistical value of peak voxel showing ALFF differences between the two groups. The positive maximum *t*-score represents an increase, and the negative maximum *t*-score represents a decrease. Abbreviations: MNI = Montreal Neurological Institute Coordinate System or Template; PCu = precuneus; ACC = anterior cingulate cortex; Ins = insula; Hip = hippocampus; BA = Brodmann's area.

## Discussion

This is the first study to investigate SIVD-related changes in intrinsic, spontaneous brain activity by measuring ALFF values from rs-fMRI signals. Previously, independent component analysis and graph analyses have been widely used in resting-state fMRI [20], [21]. However, these methods investigate resting-state fMRI signal from the aspect of temporal correlation, not from the aspect of regional activity during a resting-state [7]. Although a result of abnormal network is comprehensive and integrative, one could not draw any conclusion about which area is abnormal [6]. As for ALFF, it has been proved effectively in detecting regional spontaneous neuronal activity alteration both in animal [22] and human studies [23]–[25]. In this study, compared to controls increased ALFF values were observed in the bilateral ACC, left insula, and left hippocampus of SIVD patients. Negative correlations between the ALFF values of the left hippocampus and insula and the MMSE and MoCA scores were also found. These findings suggest that the brain regions described above may be the most affected areas in SIVD and that the increased spontaneous ALFF values of the rs-fMRI signals is a possible characteristic of cognitive impairment. The measurement of ALFF values in these regions might have great potential value in the diagnosis of SIVD. The ACC is important in attention-related cognitive and emotional processing [26]. ACC dysfunction has been reported in SIVD patients during Stroop task performance [27]. Decreasing ACC activation predicts poor attention control and a propensity for errors [28], [29]. The hippocampus plays key roles in episodic and autobiographical memory, and it is extremely sensitive to excitotoxicity from vascular disease-associated hypoxia and ischemia [30]. Autopsy [31] and neuroimaging [32], [33] studies have demonstrated that lesions in the hippocampus are common in VaD patients. The human insula is hidden deep in the cerebral hemisphere covered by the frontal and temporal opercula. It contains several functional regions involved in attention, language, speech, working memory, and memory [34].

Decreased gray matter (GM) volume in the frontal and temporal regions as well as the hippocampus and anterior cingulate cortex in SIVD patients has been previously demonstrated [11], [12]. In this study, after including GM volume as an additional covariate, the ALFF group difference exhibited highly similar spatial patterns to those for which the GM volume was not corrected. These data indicate that the increased ALFF values observed in the rs-fMRI signals were not a result of brain atrophy but an independent biomarker for SIVD. In previous studies, higher resting-state activation has also been observed in other types of dementia [8]. For example, increased spontaneous function connectivity in the precuneus, anterior insula, medial prefrontal cortex, and posterior cingulate has been found in frontotemporal dementia [35], [36]. Increased spontaneous activities in the prefrontal cortex, superior frontal gyrus, left hippocampus, and left temporal lobe were also reported in AD [8], [37], [38]. The increased activity has been explained as reflecting compensatory reactions that allow increased recruitment of neurons to compensate for neuron damage [8], [37], [39] or nonselective recruitment, which would reflect a generalized nonfunctional spread of activity [40], [41].

In this study, significantly decreased ALFF values were observed in the bilateral precuneus of SIVD patients. It showed no correlation with cognitive performance. However, it should be noted that our current data cannot differentiate neural and vascular effects. SIVD has been proved substantial CBF reductions in both frontal and parietal cortices [42], [43], which may influent cortical ALFF greatly [44], [45]. So the vascular disease in our patients may partly contribute to the results of bilateral precuneus ALFF decreasing. A previously reported PET study demonstrated hypometabolism in the precuneus of SIVD patients [46]. Structural neuroimaging studies have also demonstrated that this region presents cortical thinning in SIVD patients [12]. The precuneus is located on the medial aspect of the parietal lobe and has a number of different functions. It has been thought to be the hub of the fronto-parietal Central-executive network (CEN) [47], which is crucial for actively maintaining and manipulating information in the working memory. Margulies found that the precuneus has three discrete regions that participate in distinct functional networks [48]. The anterior precuneus connects primarily to sensorimotor areas and projected to the insula. The central precuneus is connected to the ACC and the hippocampus. The posterior precuneus connects to adjacent regions of the visual cortex. The common symptoms of SIVD, including psychomotor slowness, executive dysfunction, attention impairment, depressive mood, and unsteady gait, match symptoms that arise from disruptions in the precuneus and its projected areas (the ACC, insula, and hippocampus).

The current study has some limitations. First, the study is limited by a relatively small sample size for patients with SIVD. Further studies with larger sample sizes will be necessary to confirm the current results. Second, due to the cross-sectional group data, we could not observe dynamic ALFF changes in different courses of SIVD. Third, it should be noted that our subjects were not pathologically confirmed and thus misdiagnosis cannot be ruled out, although we employed a range of neuropsychological tests to ensure the higher specificity. Fourth, we assume the signal variation to be determined by neurophysiology/activity directly and did not consider the contribution of variability in vascular function. Lastly, although GM volumes have been applied as covariates, structural differences between groups could remain influence rs-fMRI signal measurement [49]. However, a standard data processing pipeline was followed with popular software and procedures, and most of the findings are therefore likely validated on the basis of these analyses.

## Conclusion

This is the first study to investigate SIVD-related changes in intrinsic, spontaneous brain activity by measuring ALFF values using rs-fMRI signals. Significant ALFF decreasing was found in the bilateral precuneus, and ALFF increasing was observed in the bilateral ACC, left insula and hippocampus. The ALFF values of the left insula and hippocampus exhibited negative correlations with cognitive performance. These findings suggest a spontaneous aberrant activity characteristic of SIVD and that measurement of the ALFFs in the above regions may aid in its detection.
